# Ion Migration in Two-Dimensional Organic–Inorganic Hybrid Perovskite Heterostructures: Interface Evolution, Migration Mechanisms and Device Implications

**DOI:** 10.3390/nano16110696

**Published:** 2026-06-03

**Authors:** Zhendong Weng, Junxiong Liu, Kexin Liu, Yingjie Zhou, Yaqi Zhang, Muzi Yang, Jian Chen, Weiguang Xie

**Affiliations:** 1 School of Chemistry, Sun Yat-sen University, Guangzhou 510006, China; wengzhd@mail2.sysu.edu.cn; 2 Instrumental Analysis & Research Center, Sun Yat-sen University, Guangzhou 510275, China; yangmz6@mail.sysu.edu.cn; 3 Siyuan Laboratory, Guangdong Provincial Engineering Technology Research Center of Vacuum Coating Technologies and New Energy Materials, Department of Physics, Jinan University, Guangzhou 510632, China; liujunxiong@stu.jnu.edu.cn (J.L.); kexinliu@stu2023.jnu.edu.cn (K.L.); vnwcj@stu2024.jnu.edu.cn (Y.Z.); yaqizhang1028@stu2024.jnu.edu.cn (Y.Z.)

**Keywords:** two-dimensional organic–inorganic hybrid perovskites, ion migration, perovskite heterostructures, interface evolution, spacer-cation engineering, optoelectronic devices

## Abstract

Two-dimensional organic–inorganic hybrid perovskite (2D-OIHP) heterostructures provide a versatile platform for crystal engineering because their composition, dimensionality, excitonic structure and interfacial energy alignment can be tuned at the molecular level. However, the same ionic softness that enables facile chemical transformation also leads to ion migration under thermal, electrical and optical stimuli. In 2D-OIHP heterostructures, ion migration is not only a degradation pathway; it determines whether a heterointerface remains sharp, becomes compositionally graded, evolves into a mixed-halide alloy, or forms a bias-programmed functional junction. This review summarizes recent progress in understanding ion migration in 2D-OIHP-based heterostructures, with emphasis on migration species, driving forces, pathways and interface evolution. We first classify representative fabrication strategies according to the initial interface profiles they generate. We then discuss thermally driven in-plane and out-of-plane halide migration, spacer-cation engineering for suppressing interdiffusion, and electric-field-induced directional migration in functional devices. Finally, we extract design rules and unresolved challenges for achieving stable, sharp or dynamically programmable perovskite heterostructures. The aim is to provide a mechanistic framework for using ion migration as both a stability criterion and a crystal-engineering tool in layered hybrid perovskites.

## 1. Introduction

Since the emergence of two-dimensional layered materials, their unique stacking characteristics and weak interlayer interactions have provided new opportunities for constructing van der Waals heterostructures without the strict lattice-matching requirements of conventional semiconductor heteroepitaxy [1,2]. However, several prototypical two-dimensional materials, such as graphene [3], black phosphorus [4,5] and transition-metal dichalcogenides [6,7,8], still face limitations in bandgap tunability, environmental stability or scalable heterostructure construction [9]. These limitations have motivated the exploration of alternative layered semiconductors with greater structural and compositional flexibility. In parallel, emerging two-dimensional semiconductors, such as Bi_2_O_2_Se, have also attracted attention because their structure-dependent electronic properties and tunability provide additional opportunities for next-generation electronics and optoelectronics [10].

Two-dimensional organic–inorganic hybrid perovskites (2D-OIHPs) have emerged as an important class of layered semiconductors by combining the excellent optoelectronic properties of three-dimensional halide perovskites with the structural diversity of molecularly engineered layered materials. With the rapid development of hybrid organic–inorganic halide perovskites in solar cells [11,12], light-emitting diodes [13], photodetectors [14,15] and other optoelectronic devices, 2D-OIHPs have attracted increasing attention. Their general structure consists of inorganic metal–halide octahedral slabs separated by organic spacer cations, allowing the quantum-well thickness, organic spacer, metal cation and halide composition to be tuned at the molecular level. This structural flexibility enables broad modulation of bandgap, exciton binding energy, dielectric confinement, lattice distortion and environmental stability, making 2D-OIHPs promising candidates for tunable optoelectronic materials and devices [16,17,18].

Recent progress in crystal growth, vapor-phase conversion, solution processing, ion exchange and van der Waals epitaxy has enabled the construction of lateral and vertical 2D-OIHP heterostructures [19,20,21,22,23,24,25]. These heterostructures provide additional freedom for regulating interfacial band alignment, charge separation, exciton transport and light emission. However, unlike conventional covalent semiconductors, halide perovskites possess soft ionic lattices, low ion-migration barriers and low defect formation energies [26,27,28,29,30]. The term “soft ionic lattice” describes the relatively deformable metal–halide framework of hybrid perovskites, in which partially ionic Pb–X bonds, anharmonic lattice vibrations and dynamic organic–inorganic interactions allow local structural distortions with low energetic cost. Such lattice softness is closely associated with low defect formation energies and low ion-migration barriers, enabling halide vacancies and mobile ions to redistribute under thermal, optical or electrical stimuli. In 2D-OIHPs, organic spacer layers can increase the effective migration barrier and provide directional confinement, but the inorganic slabs still retain a defect-tolerant and dynamically deformable ionic framework. As a result, mobile ions, especially halide ions and related vacancies, can migrate under thermal gradients [31], electrical bias [32,33] or other external stimuli. In heterostructures, such ion migration can directly alter the interfacial composition and therefore strongly influence band alignment, defect distribution, interface sharpness and device performance [34,35].

Despite these advances, ion migration in 2D-OIHP heterostructures has not yet been reviewed as an independent and interface-governing phenomenon. Existing reviews have mainly focused on the synthesis, crystal structures, optoelectronic properties and device applications of 2D hybrid perovskites [21,22,26,36], whereas ion migration has often been discussed as a general degradation mechanism or as a phenomenon inherited from three-dimensional perovskites [28,29,37]. In 2D-OIHP heterostructures, however, ion migration plays a more specific role: it determines whether a heterointerface remains sharp, becomes compositionally graded, evolves into a mixed-halide alloy, or forms a bias-programmed functional junction [32,38,39,40]. Therefore, understanding ion migration in these systems is essential not only for suppressing instability, but also for designing heterostructures with programmable interface composition, band alignment and device functionality.

In this review, 2D-OIHP heterostructures refer to heterointerfaces in which at least one component is a layered organic–inorganic hybrid perovskite with quantum-confined inorganic slabs separated by organic spacer cations [36,38,41,42]. The main focus is placed on all-perovskite lateral and vertical heterostructures, where ion migration directly changes interfacial composition and optoelectronic response [23,24,38,39,40]. Mixed-dimensional systems, such as 2D-OIHPs/carbon nanotube heterostructures, are discussed only when ion migration in the perovskite component directly modulates device operation [43]. Both in-plane and out-of-plane migration are included because they lead to distinct interface-evolution pathways [32,38,40]. By contrast, general synthesis of 2D perovskites, twist-angle engineering and stacking-angle-dependent physics are not treated as independent topics unless they are directly related to ion migration, interfacial alloying or migration-controlled device function.

This review first summarizes representative fabrication strategies and the initial interface profiles they generate. It then discusses thermally driven ion migration, molecular engineering for suppressing or programming ion migration, and electric-field-induced migration in functional devices. Finally, design principles and future challenges for stable and programmable 2D-OIHP heterostructures are discussed.

## 2. Fabrication Strategies and Initial Interface Profiles of 2D-OIHP Heterostructures

2D-OIHPs are typically categorized into Ruddlesden-Popper (RP) and Dion-Jacobson (DJ) phases [41]. The RP phase is described by the formula (RNH_3_)_2_A*_n_*_−1_M*_n_*X_3*n*+1_, where ${RNH}_{3}^{+}$ is a bulky organic spacer cation, A^+^ is a small monovalent cation, such as MA^+^, FA^+^ or Cs^+^, M^2+^ is a divalent metal cation, such as Pb^2+^ or Sn^2+^, X^−^ is a halide anion, and *n* is the number of corner-sharing MX_6_ octahedral layers between organic spacer sheets [26,36]. Unlike DJ-phase perovskites with more strongly connected interlayer structures, RP-phase perovskites feature van der Waals interfaces between adjacent layers without surface dangling bonds. This eliminates the need for lattice matching, enabling their exfoliation into monolayers and subsequent restacking into heterostructures [42,44].

In contrast, DJ-type perovskites contain divalent spacer cations that bridge adjacent inorganic slabs more strongly, leading to reduced interlayer spacing and modified lattice rigidity. These structural differences are important for ion migration because the migration pathway, vacancy formation energy and interfacial ion diffusion are strongly affected by interlayer spacing, organic–inorganic coupling and lattice softness. By selecting appropriate layered phases and designing suitable interfacial band alignment, charge generation and transport in 2D-OIHP heterostructures can be effectively regulated [38].

The fabrication route determines not only the geometry of 2D-OIHP heterostructures, but also the initial interfacial composition, defect distribution and possible migration pathway. Therefore, representative fabrication methods are discussed here according to the interfacial profiles they generate, including composition-gradient interfaces formed by ion exchange, vertically separated interfaces formed by sequential crystallization, sharp van der Waals interfaces produced by epitaxial growth and transfer-stacked interfaces used for device studies. The relationship between fabrication strategy and ion-migration relevance is summarized in Table 1.

**Figure 1:**
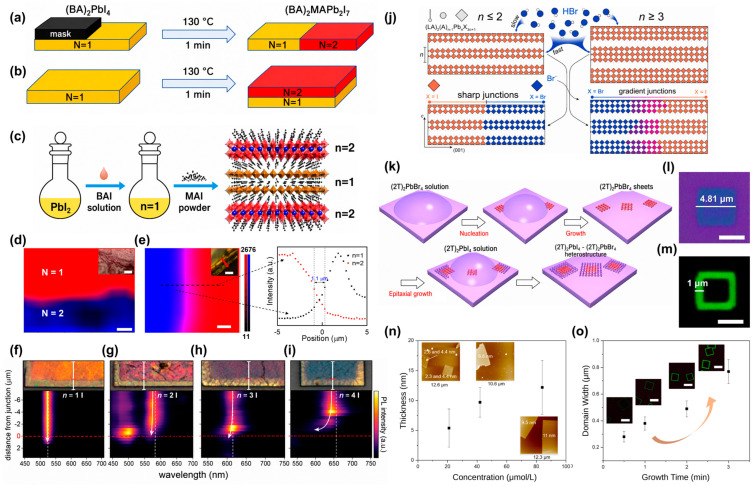
Fabrication strategies and interface characterization of 2D-OIHP heterostructures. (**a**,**b**) Schematic synthesis of (BA)_2_PbI_4_ (*n* = 1)/(BA)_2_MAPb_2_I_7_ (*n* = 2) lateral and vertical heterostructures by gas–solid intercalation [24]. Copyright 2017, American Chemical Society. (**c**) Solution-processed synthesis of (BA)_2_PbI_4_/(BA)_2_MAPb_2_I_7_ heterostructures [23]. Copyright 2019, American Chemical Society. (**d**) PL mapping image of the junction regions of a lateral (BA)_2_PbI_4_/(BA)_2_MAPb_2_I_7_ heterostructure [24]. Copyright 2017, American Chemical Society. (**e**) Spatially resolved cross-section PL mapping at 520 and 590 nm for the (BA)_2_PbI_4_/(BA)_2_MAPb_2_I_7_ heterostructure [23]. Copyright 2019, American Chemical Society. (**f**–**i**) PL line scans collected along 12 μm scan paths across the lateral heterointerfaces of (HA)_2_(MA)*_n_*_−1_Pb*_n_*I_3*n*+1_ perovskites with different inorganic-layer thicknesses (*n* = 1–4) after HBr-vapor anion exchange. (**j**) Schematic comparison of anion-exchange processes at RP perovskite lateral heterojunctions with different *n* values [39]. Copyright 2021, American Chemical Society. (**k**) Epitaxial growth of (2T)_2_PbI_4_-(2T)_2_PbBr_4_ lateral heterostructures. (**l**) Optical and (**m**) photoluminescence images of a (2T)_2_PbI_4_-(2T)_2_PbBr_4_ lateral heterostructure. (**n**) Precursor solution concentration dependence of (2T)_2_PbBr_4_ sheets thickness. (**o**) Lateral size of (2T)_2_PbI_4_ domains with respect to the growth time [44]. Copyright 2020, Springer Nature Limited.

Gas–solid intercalation and ion exchange were among the earliest methods used to create 2D-OIHP heterostructures. As shown in Figure 1a,b, a preformed (BA)_2_PbI_4_ crystal can be exposed to MAI or MACl vapor through a mask or from the top side, allowing MA^+^ to diffuse into the layered crystal and partially replace BA^+^. This process converts part of the *n* = 1 RP phase into a higher-*n* phase, forming lateral or vertical (BA)_2_PbI_4_/(BA)_2_MAPb_2_I_7_ heterostructures [24]. The spatially resolved PL mapping in Figure 1d shows that the emission peak of 514 nm at the red *n* = 1 area gradually shifts to 570 nm at the blue *n* = 2 area, indicating that ion exchange produces a compositionally graded interface rather than an ideally abrupt boundary. Thus, the figure illustrates both the advantage and limitation of ion-exchange fabrication: it is simple and versatile, but the final interface sharpness is difficult to control precisely.

Solution-phase sequential crystallization provides another route for constructing 2D-OIHP heterostructures. As illustrated in Figure 1c, BAI solution was injected into a PbI_2_ solution at 80 °C, followed by the addition of MAI precursor. Due to solubility differences, (BA)_2_PbI_4_ crystals (*n* = 1) preferentially formed as the temperature decreased, followed by the precipitation of (BA)_2_MAPb_2_I_7_ crystals (*n* = 2). Due to lattice mismatch in the lateral direction, perovskite phases with different *n* values preferentially grew vertically rather than laterally. The PL cross-section mapping in Figure 1e shows distinct *n* = 1 (blue) and *n* = 2 (red) emission regions separated by a transition region, again revealing that interface formation and ion redistribution are coupled during growth [23]. This example is important because it demonstrates that interface width is determined not only by post-growth diffusion, but also by crystallization sequence and solubility difference during heterostructure formation.

Halide vapor exchange further reveals how the effective dimensionality and *n* value regulate ion migration in RP perovskite heterostructures. As shown in Figure 1f–i, PL line scans across RP perovskite heterointerfaces indicate that higher-*n* phases exhibit more gradual I^−^/Br^−^ interdiffusion and continuous emission shifts, whereas lower-*n* phases preserve sharper lateral interfaces after vapor exchange [39]. The mechanism summarized in Figure 1j suggests that this *n*-dependent behavior originates from the different degrees of inorganic-slab continuity and organic-spacer confinement. In low-*n* RP structures, the inorganic Pb–X framework is thinner and more frequently separated by insulating organic spacer layers, leading to stronger dimensional/dielectric confinement and a higher effective barrier for vacancy-mediated halide migration. These structural features suppress interfacial Br^−^/I^−^ interdiffusion and help maintain abrupt heterointerfaces. In contrast, higher-*n* RP phases contain thicker and more continuous inorganic slabs, which provide more extended diffusion pathways and gradually approach a more 3D-like ion-transport behavior, resulting in broader compositionally graded interfaces. This comparison provides a clear structural explanation for why low-*n* 2D-OIHPs can suppress ion migration more effectively than high-*n* or 3D-like perovskite phases. Therefore, the *n* value should be considered an important structural parameter for programming interfacial ion migration in 2D-OIHP heterostructures.

Ion diffusion and exchange provide simple and versatile routes for constructing 2D-OIHP heterostructures, but they often produce compositionally graded interfaces that are difficult to control precisely. In comparison, low-temperature van der Waals epitaxy offers an alternative strategy for producing sharper lateral interfaces, especially in layered material systems with weak interlayer interactions [2,19,35]. As schematically shown in Figure 1k, preformed (2T)_2_PbBr_4_ nanosheets were first obtained by a solvent-evaporation method. After lowering the growth temperature, a precursor solution containing the iodide perovskite component was added, allowing (2T)_2_PbI_4_ to nucleate and crystallize along the edges of the central (2T)_2_PbBr_4_ nanosheets. The optical image in Figure 1l and the corresponding PL image in Figure 1m show a well-defined bromide domain surrounded by an iodide domain, confirming the formation of a lateral (2T)_2_PbBr_4_-(2T)_2_PbI_4_ heterostructure with a relatively sharp interface [44]. This interface is much more clearly defined than the compositionally graded boundaries commonly produced by uncontrolled ion-exchange processes. Moreover, the structural dimensions of the heterostructures can be tuned by growth parameters. As shown in Figure 1n, the thickness of (2T)_2_PbBr_4_ sheets increases with precursor concentration, whereas Figure 1o shows that the lateral size of the (2T)_2_PbI_4_ domain increases with growth time. These results indicate that van der Waals epitaxy not only enables the construction of lateral 2D-OIHP heterostructures, superlattices and multi-heterostructures, but also provides a way to regulate interface geometry and the characteristic length scale over which subsequent ion migration may occur [44].

However, from a practical device-integration perspective, the scalability and manufacturability of 2D-OIHP heterostructures remain nontrivial. Ion-exchange and vapor-assisted methods are versatile, but their interface width and composition gradient are sensitive to processing time, precursor concentration and local defect density. Solution-processed sequential crystallization is more compatible with large-area fabrication, yet controlling nucleation, grain boundaries and vertical phase distribution remains challenging. In contrast, epitaxial growth and dry-transfer stacking can generate sharper interfaces, but they often suffer from limited throughput, substrate constraints, interfacial contamination or transfer-induced gaps. Therefore, future studies should evaluate large-area uniformity, processing yield, contact compatibility, encapsulation and long-term operational stability in addition to heterointerface formation. These issues are also consistent with broader challenges in modern two-dimensional heterostructures, where scalable fabrication, interface cleanliness, stacking precision and reproducible device integration remain important factors affecting device performance and application potential [45].

## 3. Thermally Driven Ion Migration in 2D-OIHP Heterostructures

Thermally driven ion migration in 2D-OIHP heterostructures is generally initiated by concentration gradients, defect-assisted diffusion and lattice softening at elevated temperature. Unlike bulk 3D perovskites, the layered architecture of 2D-OIHPs introduces strong anisotropy: in-plane migration can broaden lateral interfaces, whereas out-of-plane migration is strongly affected by organic spacer layers and interlayer barriers. Therefore, thermal ion migration should be discussed not only in terms of activation energy, but also in terms of migration direction, interface geometry and dimensional confinement.

Compared with conventional 3D perovskites, the layered structure of 2D-OIHPs changes ion migration into a more anisotropic and interface-sensitive process. In 3D hybrid lead-halide perovskites, vacancy-assisted ionic transport has been widely recognized as an important mechanism underlying mixed ionic–electronic conduction [46]. In 2D-OIHPs, organic spacer layers interrupt out-of-plane connectivity and increase the effective barrier for interlayer transport, but they do not completely eliminate ion migration, especially under thermal, optical or electrical stimuli [47]. Therefore, ion migration and interfacial stability in 2D-OIHP heterostructures are governed by the combined effects of spacer-mediated confinement, defect-assisted pathways, migration direction and interface geometry.

Generally, smaller ions induce less lattice distortion during diffusion and are therefore more prone to migrate. Ion migration in halide perovskites is closely related to defect formation because point defects provide pathways for mobile ions. Typical point defects include Schottky defects, Frenkel defects, halide vacancies and interstitial ions [28,37]. Schottky defects involve the simultaneous formation of cation and anion vacancies to maintain charge neutrality, whereas Frenkel defects consist of a vacancy–interstitial pair generated by the displacement of an ion from its lattice site. These defects can be formed during crystal growth or activated under thermal, optical or electrical stimuli. When the defect formation energy and migration activation energy are low, mobile ions can redistribute more readily, leading to local compositional changes, charge accumulation and defect redistribution [28,37,48]. In 2D-OIHPs, organic spacer layers usually increase the effective migration barrier and suppress out-of-plane ion transport, but they do not completely eliminate halide motion, especially at edges, grain boundaries and heterointerfaces.

Building on this defect-mediated migration picture, vacancy-mediated transport is frequently invoked to explain ion redistribution in 2D-OIHP heterostructures. However, the actual vacancy concentration is rarely measured directly. First-principles calculations can provide vacancy formation energies, migration barriers and diffusion-related energetics, which help assess the thermodynamic feasibility of vacancy-assisted ion migration [49]. However, direct experimental quantification of vacancy concentration in 2D-OIHP heterostructures remains largely absent. Most existing discussions infer vacancy-mediated transport indirectly from calculated defect energetics, ion-conductivity behavior, activation energies, or spatially resolved ion redistribution, rather than from measured vacancy densities. Therefore, in current studies of 2D-OIHP heterostructures, vacancy concentration should be regarded as an indirect or semi-quantitative descriptor unless supported by complementary theoretical, electrical and spatially resolved compositional evidence.

Beyond point defects, extended defects, such as grain boundaries and edges, may also play important roles in ion migration in practical 2D-OIHP heterostructure devices. Although their influence in all-2D OIHP heterostructures has not yet been systematically investigated, related studies on halide perovskites suggest that these extended defects may actively affect ion migration and device instability. In polycrystalline films, grain boundaries and edges can act as defect-rich regions containing mobile vacancies, interstitials and under-coordinated sites, thereby providing preferential pathways or accumulation sites for ionic defects. For example, Zhou et al. reported iodine-vacancy accumulation at grain boundaries and its correlation with enhanced charge recombination [50], while machine-learning force-field simulations further identified grain boundaries as energetically favorable ion-migration channels [51]. Spatially resolved scanning-probe measurements also showed heterogeneous local ion motion that can be strongly suppressed by surface passivation [52]. More directly related to layered heterostructures, Wu et al. demonstrated that edge-initiated in-plane cation migration can drive the lateral formation and stabilization of 3D/2D perovskite heterostructures from 2D nanoplatelets [53]. These findings suggest that grain boundaries and edge defects in practical 2D-OIHP heterostructure devices may serve as ion/vacancy migration pathways affecting interface broadening, interfacial recombination and operational stability. However, direct evidence correlating grain-boundary/edge structures with ion-migration pathways and device performance in all-2D OIHP heterostructures remains limited, calling for spatially resolved operando studies.

**Figure 2:**
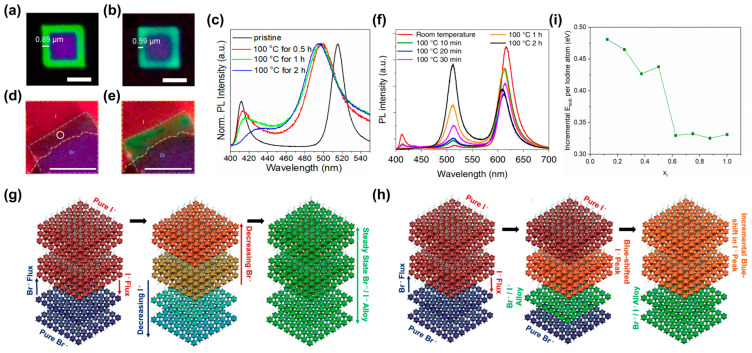
Thermally driven halide interdiffusion in lateral and vertical 2D-OIHP heterostructures. (**a**,**b**) PL images of a (BA)_2_PbI_4_–(BA)_2_PbBr_4_ lateral heterojunction in the pristine state and after heating at 100 °C for 1 h. (**c**) PL spectral changes in the lateral heterojunction before and after heating [44]. Copyright 2020, Springer Nature Limited. (**d**,**e**) PL images of a (BA)_2_PbBr_4_/(BA)_2_(MA)_2_Pb_3_I_10_ vertical heterostructure in the pristine state and after heating at 100 °C for 1 h. (**f**) PL spectral changes in the vertical heterostructure before and after heating. (**g**) Schematic concentration-gradient-driven Br/I diffusion model. (**h**) Layer-by-layer evolution of the concentration profile in the vertical heterostructure. (**i**) Calculated substitution energy of iodide ions in (BA)_2_PbBr_4_ [40]. Copyright 2021, Springer Nature Limited. Scale bars in (**a**,**b**,**d**,**e**): 3 μm.

The thermal evolution of lateral and vertical 2D-OIHP heterostructures reveals two distinct modes of halide redistribution. In the lateral (BA)_2_PbI_4_–(BA)_2_PbBr_4_ heterostructure shown in Figure 2a–c, annealing blurs the originally well-defined Br-rich/I-rich boundary and produces PL peak shifts and spectral broadening, indicating that in-plane Br^−^/I^−^ interdiffusion converts a designed sharp junction into a compositionally graded interface [44]. In contrast, in the vertical (BA)_2_PbBr_4_/(BA)_2_(MA)_2_Pb_3_I_10_ heterostructure shown in Figure 2d–f, thermal annealing leads to the emergence of an intermediate PL band rather than a simple gradual shift in the original Br-rich and I-rich emissions [40]. This behavior suggests that out-of-plane halide migration does not follow a simple continuous concentration-gradient model. As schematically shown in Figure 2g, such a model would predict a smooth Br/I composition profile across the vertical stack. However, the layer-by-layer diffusion model in Figure 2h and the calculated substitution energies in Figure 2i indicate that iodide incorporation becomes more favorable after the initial substitution step, leading to a threshold-dependent alloy-formation process. This comparison indicates that in-plane migration is mainly reflected by lateral interface broadening, whereas out-of-plane migration is more strongly affected by interlayer barriers, layer-by-layer transport and composition-dependent substitution energetics. Therefore, thermally driven ion migration in 2D-OIHP heterostructures should not be treated as a single bulk-like diffusion process, but as a direction-dependent interface-evolution pathway governed by dimensional confinement, defect distribution and organic-spacer-mediated barriers.

Recent studies have shown that long-chain organic ligands in 2D perovskites can act as effective barriers to suppress out-of-plane ion migration [54,55]. Compared with 3D perovskites, 2D perovskites generally exhibit higher vacancy formation energies for methylammonium and iodide vacancies, such as V_MA_ and V_I_, making vacancy generation less favorable [56]. The reduced concentration of available vacancy sites increases the effective ion-migration barrier and thereby enhances the intrinsic ionic and structural stability of 2D perovskites. This explains why out-of-plane migration in 2D-OIHP heterostructures is usually more strongly confined than in 3D perovskites, although halide migration can still occur at heterointerfaces, edges or defect-rich regions under thermal or electrical stimuli.

Because these migration processes are often inferred from PL changes, the interpretation of PL results should be treated carefully. A PL peak shift generally reflects a change in local bandgap, while peak broadening often indicates spatial or compositional heterogeneity within the probed region. It should be noted that PL evolution is not governed exclusively by ion migration. For example, photo-induced de-mixing in Dion-Jacobson 2D mixed-halide perovskites can generate iodide-rich and bromide-rich domains, leading to local bandgap redistribution and corresponding optical spectral changes [57]. In addition, applied stress, intrinsic strain, and pressure-dependent lattice distortion in quasi-2D or layered hybrid perovskites have been shown to modify optical responses, including PL peak position, emission intensity, lifetime, and wavelength-shifted emission [58,59,60]. These observations indicate that strain relaxation, phase segregation and structural deformation may also contribute to PL shifts in 2D-OIHP systems. Nevertheless, such effects do not invalidate the use of PL mapping for identifying ion migration. In mixed-halide perovskites, phase segregation is usually coupled with halide redistribution, and strain can also regulate ion-migration energetics rather than acting as a completely independent factor. Therefore, when PL peak shifts are spatially resolved, time-dependent, consistent with the expected Br^−^/I^−^ redistribution pathway, and accompanied by interface broadening or mixed-halide alloy emission, they can still provide strong indirect evidence for ion migration. To avoid overinterpretation, PL mapping should be correlated with complementary structural and compositional probes, such as XRD/GIWAXS, Raman spectroscopy, elemental mapping, ToF-SIMS, KPFM and in situ/operando measurements, to distinguish ion migration from pure strain relaxation, phase transition or other bandgap-changing processes.

## 4. Molecular Engineering for Suppressing and Programming Ion Migration

Molecular spacer engineering provides a rational route to regulate ion migration in 2D-OIHP heterostructures. Bulky, rigid and π-conjugated organic cations can increase steric hindrance, strengthen organic–inorganic interactions, reduce lattice softness and suppress halide interdiffusion. This strategy is especially important for maintaining sharp heterointerfaces under thermal or electrical stress. However, complete suppression of ion migration is not always desirable. For applications requiring graded band alignment or reconfigurable junctions, a moderate and controllable level of ion migration may be beneficial. Therefore, spacer design should be guided by the targeted interface profile: sharp, graded or dynamically programmable.

**Figure 3:**
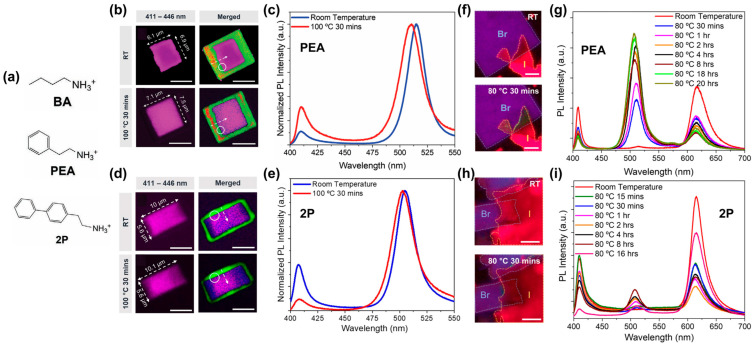
Spacer-cation-regulated halide migration in 2D-OIHP lateral and vertical heterostructures. (**a**) Molecular structures of the organic spacer cations BA, PEA and 2P. (**b**,**c**) PL confocal microscopy images and corresponding PL spectra of the (PEA)_2_PbI_4_–(PEA)_2_PbBr_4_ lateral heterostructure before and after heating at 100 °C for 30 min. (**d**,**e**) PL confocal microscopy images and corresponding PL spectra of the (2P)_2_PbI_4_–(2P)_2_PbBr_4_ lateral heterostructure under the same thermal treatment [61]. Copyright 2021, Wiley-VCH GmbH. (**f**,**g**) PL images and corresponding PL spectra of the (PEA)_2_PbBr_4_–(BA)_2_(MA)_2_Pb_3_I_10_ vertical heterostructure before and after heating at 80 °C for 30 min. (**h**,**i**) PL images and corresponding PL spectra of the (2P)_2_PbBr_4_–(BA)_2_(MA)_2_Pb_3_I_10_ vertical heterostructure under the same thermal treatment [40]. Copyright 2021, Springer Nature Limited.

Figure 3a summarizes representative organic spacer cations, including BA, PEA and 2P [61]. Compared with flexible aliphatic BA, aromatic and conjugated spacers introduce stronger structural rigidity and hydrophobicity, and they can modify Pb–I–Pb bond angles, N–I interactions and lattice distortion. In particular, extended π-conjugated organic cations have been reported to rigidify the inorganic lattice in organic semiconductor-incorporated 2D perovskites, which provides a useful structural basis for understanding spacer-regulated ion migration [62]. These molecular-level differences directly affect ion migration because the inorganic framework and organic spacer layer are mechanically and electrostatically coupled in layered perovskites.

From a mechanistic perspective, spacer-cation engineering suppresses ion migration through coupled steric and energetic effects, rather than by simple physical blocking alone. Rigid, bulky or π-conjugated spacer cations can restrict local Pb–X framework deformation and strengthen organic–inorganic coupling, which may increase the energetic penalty for mobile-defect formation and vacancy-assisted halide hopping [56]. First-principles calculations on 2D lead-halide perovskite lateral heterostructures further indicate that organic spacers can modify anionic diffusion pathways and migration energetics [49]. Therefore, the suppressed Br^−^/I^−^ interdiffusion observed in rigid-spacer-based heterostructures can be understood as a combined result of steric confinement, enhanced lattice rigidity, modified defect energetics and increased migration barriers.

The lateral heterostructures in Figure 3b–e illustrate how spacer selection regulates Br^−^/I^−^ interdiffusion under thermal stress [61]. In PEA-based A_2_PbI_4_–A_2_PbBr_4_ lateral heterostructures, thermal annealing leads to expansion of the Br-rich region and a blue shift in the iodide-rich emission, indicating interfacial halide interdiffusion and compositional redistribution. In contrast, the 2P-based heterostructure shows only minor spatial and spectral changes after the same treatment, suggesting that Br^−^/I^−^ interdiffusion is strongly suppressed by the more rigid and extended π-conjugated spacer [61]. This comparison indicates that spacer rigidity and conjugation can reduce halide mobility and help preserve lateral interface sharpness.

A similar spacer-dependent trend is observed in vertical heterostructures, as shown in Figure 3f–i [39]. When the iodide-rich layer is fixed as (BA)_2_(MA)_2_Pb_3_I_10_ and the bromide-rich layer is changed from (PEA)_2_PbBr_4_ to (2P)_2_PbBr_4_, both systems show mixed-halide alloy emission after thermal treatment. However, the alloy-phase PL is much weaker in the 2P-based heterostructure, indicating that the more rigid 2P spacer also suppresses out-of-plane Br^−^/I^−^ interdiffusion [39]. Therefore, organic spacer cations should not be regarded as passive insulating layers, but as active structural parameters that regulate ion-migration kinetics, alloy formation and heterointerface stability.

These results demonstrate that molecular spacer engineering can modulate the rate and extent of halide interdiffusion in both lateral and vertical 2D-OIHP heterostructures. In BA-, PEA- and 2P-based systems, Br^−^/I^−^ interdiffusion is progressively suppressed as the spacer becomes more rigid, bulky and π-conjugated [40,61]. From a design perspective, molecular engineering can therefore be used in two complementary ways. When the goal is to preserve a compositionally sharp or optically well-defined heterointerface, rigid and hydrophobic spacers should be selected to suppress halide interdiffusion, thereby stabilizing interface-dependent optical and electronic responses. When the goal is to create a compositionally graded interface, a tunable emission region or a programmable junction, less restrictive spacers or controlled post-treatment may be used to allow limited ion redistribution. This distinction should be made explicit in future 2D-OIHP heterostructure design.

Nevertheless, the use of rigid or bulky spacer cations to suppress ion migration should be balanced against their influence on electronic transport. Recent studies have emphasized that although 2D perovskites show improved structural stability and compositional tunability compared with 3D perovskites, their relatively poor charge transport remains a critical limitation, and organic spacer-cation engineering is one of the key strategies for addressing this issue [63]. In this context, spacer layers that effectively block halide/vacancy migration may also act as electronically insulating barriers, weaken interlayer electronic coupling and hinder out-of-plane carrier transport, especially in vertical heterostructures. Importantly, this trade-off is not necessarily unavoidable. For example, ab initio simulations have suggested that fluorine substitution of organic spacers can enhance structural stability, reduce reorganization energy and improve electron/hole mobility by one to two orders of magnitude [64]. Therefore, spacer-cation design should not be evaluated only by ion-migration suppression, but also by carrier mobility, interlayer charge transfer, charge extraction and the specific device geometry.

## 5. Electric-Field-Driven Ion Migration and Device Applications

When an electric field is applied, mobile ions in perovskites, especially halide anions such as I^−^, can migrate toward the corresponding electrodes, accompanied by the redistribution of vacancies within the soft ionic lattice [29,65]. This electric-field-driven ion migration is common in perovskite devices, particularly in photodiodes and other junction-based optoelectronic devices, where ionic redistribution can affect charge collection, carrier transport and interfacial electric fields [29,65]. The key distinction between thermal diffusion and electric-field-driven migration is that thermal diffusion is usually governed by concentration gradients and tends to broaden the interface, whereas electrical bias can generate directional, asymmetric and history-dependent ionic redistribution. This asymmetry is the basis for bias-programmed junction behavior in 2D-OIHP devices.

**Figure 4:**
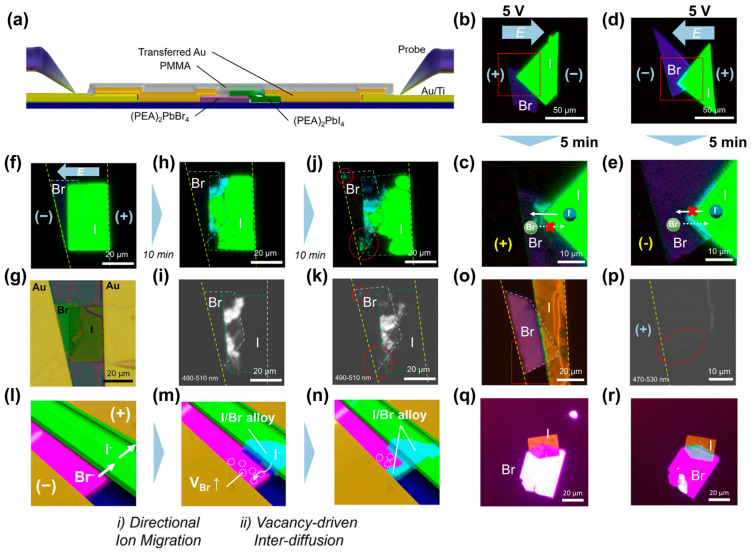
Electric-field-driven ion migration and vacancy-assisted diffusion in 2D-OIHP heterostructures. (**a**) Cross-sectional schematic of the (PEA)_2_PbI_4_–(PEA)_2_PbBr_4_ heterojunction device. (**b**,**d**) Original photoluminescence (PL) images of the (PEA)_2_PbI_4_–(PEA)_2_PbBr_4_ heterojunction before applying electrical bias. (**c**,**e**) PL images of the (PEA)_2_PbI_4_–(PEA)_2_PbBr_4_ heterojunction after applying a 5 V bias for 5 min with the bias direction toward the I-rich side (**c**) and the Br-rich side (**e**), respectively. (**f**,**g**) PL and optical images of the pristine (PEA)_2_PbI_4_–(PEA)_2_PbBr_4_ heterojunction. (**h**,**j**) PL images after applying a 20 V bias toward the (PEA)_2_PbI_4_ side for 10 and 20 min, respectively. (**i**,**k**) Confocal PL images collected in the 490–510 nm range corresponding to panels (**h**,**j**). (**l**–**n**) Schematic mechanisms of field-driven directional ion migration and vacancy-driven mutual diffusion. (**o**,**p**) PL and 490–510 nm confocal PL images of the (PEA)_2_PbBr_4_–(PEA)_2_(MA)Pb_2_I_7_ heterojunction after applying a 3 V bias toward the (PEA)_2_(MA)Pb_2_I_7_ side for 5 min. (**q**,**r**) PL images of the same heterojunction before and after thermal annealing at 110 °C for 2 h [32]. Copyright 2024, Elsevier Inc.

To investigate electrically induced ion migration in 2D perovskites, a single-crystalline (PEA)_2_PbI_4_–(PEA)_2_PbBr_4_ heterostructure device with van der Waals contact was constructed, enabling in situ PL imaging under electrical bias, as shown in Figure 4a [32]. In the initial PL images, the purple and green regions correspond to (PEA)_2_PbBr_4_ and (PEA)_2_PbI_4_, respectively, with an overlapping heterointerface region (Figure 4b,d). After applying a 5 V bias toward the iodide-rich side for 5 min, a sky-blue emission region appears near the overlap region (Figure 4c), indicating the formation of an I/Br mixed-halide alloy caused by I^−^ migration into the bromide-rich component [32]. When the bias direction is reversed, the PL change is much weaker (Figure 4e), which may arise from weaker optical contrast associated with Br^−^ migration into the iodide-rich region rather than the absence of Br^−^ migration. In mixed-halide perovskites, iodide-rich domains usually have lower bandgaps and can dominate the PL response, which has also been widely observed in studies of mixed-halide perovskite phase redistribution and PL evolution [66,67]. Therefore, the asymmetric PL response should be understood as a combined result of ion-migration direction, bandgap contrast, PL quantum yield and vertical stacking order.

Under stronger and prolonged electrical stimulation, the migration behavior becomes more complex. As shown in Figure 4f–k, applying a 20 V bias induces boundary displacement and sky-blue alloy-phase emission localized near the heterointerface, while reversed bias over longer durations can also generate alloy emission on the bromide-rich side [32]. This behavior suggests that ion redistribution cannot be explained solely by unidirectional halide drift. A two-stage mechanism has therefore been proposed, as illustrated in Figure 4l–n: directional halide migration first follows the applied electric field, while sustained bias leads to vacancy accumulation and a vacancy concentration gradient that can further drive defect-assisted mutual diffusion [32]. This picture is consistent with previous studies showing that ionic motion under bias is strongly coupled with defect redistribution, vacancy migration and electrochemical ion accumulation [28,53,54].

These observations indicate that electric-field-induced migration in 2D-OIHP heterostructures is governed not only by field direction and strength, but also by vacancy dynamics, heterointerface structure and local defect distribution. Such coupled ionic processes are important for understanding bias-induced instability, interfacial alloying and reconfigurable optoelectronic behavior in halide perovskite heterostructures [68,69].

Edge effects further distinguish electric-field-driven migration from thermally driven diffusion. In the (PEA)_2_PbBr_4_–(PEA)_2_(MA)Pb_2_I_7_ heterostructure, applying a 3 V bias for 5 min toward the (PEA)_2_(MA)Pb_2_I_7_ side induces alloy-phase formation exclusively along the junction edge, as shown in Figure 4o,p. This localized alloy formation suggests directional migration of iodide anions toward the (PEA)_2_PbBr_4_ region through edge-associated pathways. By contrast, after thermal annealing at 110 °C for 2 h, a more uniform sky-blue alloy phase forms across the junction region (Figure 4q,r), indicating broader concentration-gradient-driven anion diffusion. Therefore, electrical bias can activate preferential ion-migration channels at conductive or defect-rich edges, while thermal diffusion tends to produce more spatially extended interfacial alloying [32].

**Figure 5:**
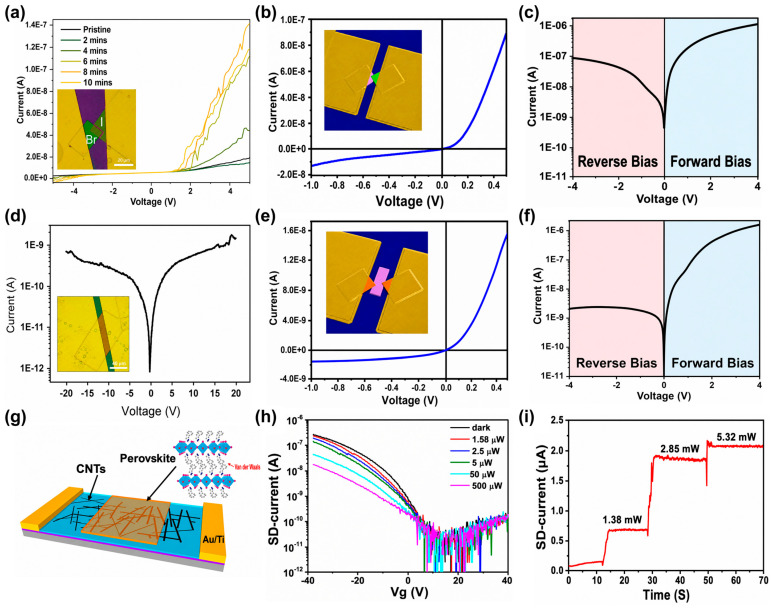
Ion-migration-modulated electrical and photoresponse characteristics in 2D-OIHP heterostructure devices. (**a**) Evolution of current–voltage (I-V) curves with bias duration in the single-heterojunction device; inset: optical image of the device. (**b**,**c**) Current–voltage (I-V) curves of the single-heterojunction device plotted on linear and logarithmic y-axis scales, respectively. (**d**) Current–voltage (I-V) curves of a non-heterojunction single-crystalline flake device plotted on a logarithmic y-axis scale. (**e**,**f**) Current–voltage (I-V) curves of the dual-heterojunction device plotted on linear and logarithmic y-axis scales, respectively; insets in (**b**,**e**) show schematic diagrams of the single- and dual-heterojunction devices [32]. Copyright 2024, Elsevier Inc. (**g**) Device schematic of the 2D perovskite/carbon nanotube (CNT) heterojunction transistor. (**h**) Source–drain (SD) current of the perovskite/CNT heterojunction measured in a vacuum chamber. (**i**) Current–time (I-T) curves of the device under different photoresponse conditions [43]. Copyright 2019, American Chemical Society.

The device consequences of field-driven migration are summarized in Figure 5. As shown in Figure 5a, when a sustained 3 V bias is applied to the single-heterostructure device, the forward current increases more significantly than the reverse current. Figure 5b,c further show that the initially nearly symmetric current–voltage response gradually evolves into clear rectifying behavior, with a rectification ratio of approximately 10 after prolonged biasing. By contrast, Figure 5d shows that the non-heterojunction control device based on a single-crystal flake maintains nearly symmetric I-V characteristics under comparable bias conditions, confirming that the diode-like response originates mainly from bias-induced ion redistribution at the heterointerface rather than from the intrinsic electrode contact alone [32].

This behavior can be understood as a consequence of sustained directional halide migration, which creates an asymmetric distribution of mobile ions, vacancies and interstitial defects across the junction. The resulting defect asymmetry can modify the local carrier concentration and interfacial band bending, producing a self-doping-like effect and an effective p–n-junction-like electronic asymmetry [70,71,72]. In addition, the current asymmetry between forward and reverse bias may also be assisted by the bandgap difference between the two components of the heterostructure. Similar ion-migration-induced polarization effects have been widely discussed in 3D halide perovskites, but the layered structure of 2D-OIHPs and the organic spacer-cation barrier may help confine ion redistribution and stabilize the programmed junction state, thereby maintaining diode-like characteristics during repeated bias cycling [33,73].

Heterostructure geometry further amplifies this migration-induced electronic asymmetry. As shown in Figure 5e,f, the dual-junction device exhibits a much higher rectification ratio under sustained 4 V bias applied to the Br-rich side, reaching up to 10^3^. This value is markedly higher than that of the single-heterostructure device, indicating that increasing the number of heterointerfaces can strengthen the accumulated ionic and electronic imbalance and thereby enhance rectification. Therefore, field-driven ion migration in 2D-OIHP heterostructures should not be regarded only as a degradation pathway; when spatially controlled, it can also be used to construct reconfigurable diode-like optoelectronic functions [32].

Beyond all-perovskite devices, ion migration can also modulate mixed-dimensional interfaces. As shown in Figure 5g–i, a 2D perovskite/carbon nanotube heterostructure field-effect transistor was constructed to probe photoinduced ionic redistribution in 2D perovskite flakes. Because single-walled carbon nanotubes are highly sensitive to local electrostatic potentials, they can serve as an electrical probe for monitoring ion distribution near the perovskite/CNT interface. Under illumination, the source–drain current decreases with increasing light intensity, showing a distinct negative photogating effect rather than direct photocurrent injection from the 2D perovskite to the CNT channel [43]. This behavior indicates that photoinduced ion migration or ionic redistribution in the 2D perovskite generates a local interfacial gating effect, thereby modulating the CNT channel conductance. Although this device is not an all-perovskite heterostructure, it is relevant to this review because it demonstrates how ion migration in 2D-OIHPs can be converted into a measurable electrical response in mixed-dimensional heterostructures.

Electrically induced ion migration has been shown to enable device-level modulation in 2D-OIHP heterostructures. For example, directional halide redistribution in 2D perovskite heterostructures can induce interfacial alloying and bias-programmed rectifying behavior, indicating that ionic redistribution can effectively modify interfacial barriers and charge-transport characteristics [32]. More broadly, ion migration and resistive switching in metal–halide perovskites have also been exploited for neuromorphic and memristive applications. For example, a lead-free Cs_3_Bi_2_I_6_Br_3_ halide perovskite memristor has been reported to emulate biologically inspired spike-timing-dependent plasticity, including long-term potentiation, long-term depression and synaptic memory consolidation [74]. Although such memristive devices are beyond the main scope of this review, they highlight a broader implication of controlled ionic redistribution: ion migration can be harnessed not only for reconfigurable optoelectronic responses, but also for artificial synaptic and memory-like functionalities.

However, the long-term impact of ion migration on device reliability remains insufficiently understood. Systematic reliability metrics, including retention stability after programming, endurance under repeated bias cycling, hysteresis evolution, switching reproducibility, device-to-device variation and long-term operational degradation, have not yet been fully evaluated for 2D-OIHP heterostructure devices. Recent studies on related perovskite memory systems provide useful benchmarks: mixed-dimensional perovskite heterostructure memories show improved endurance, retention, cycle-to-cycle reproducibility and storage stability [75], while 2D perovskite memristors, Ruddlesden–Popper ReRAM devices and Dion-Jacobson synaptic arrays exhibit promising retention, cycling endurance, switching reproducibility, device yield and moisture stability [76,77,78]. Nevertheless, these examples are not all based on lateral or vertical 2D-OIHP heterostructures. Therefore, direct evidence linking ion-migration pathways to retention stability, hysteresis evolution, switching reproducibility and long-term operational degradation in 2D-OIHP heterostructure devices remains limited.

Taken together, these examples indicate that ion migration is a double-edged phenomenon in 2D-OIHP-related heterostructures. Uncontrolled migration can degrade interface sharpness and device stability, whereas controlled migration can enable reconfigurable diodes, phototransistors and memory-like optoelectronic responses. Future device studies should therefore move beyond initial PL or I-V modulation and quantitatively correlate ion redistribution with migration reversibility, retention time, cycling fatigue, hysteresis evolution, environmental stability and long-term structural or chemical changes under realistic operating conditions.

Overall, ion migration in 2D-OIHP heterostructures is controlled by the combined effects of external stimulus, mobile species, migration pathway and interface/device response. Table 2 summarizes representative migration behaviors discussed in this review, including thermal halide interdiffusion, spacer-cation-mediated suppression, electric-field-induced directional and vacancy-mediated migration, and photo-assisted migration in CNT/2D-OIHP devices. The comparison highlights that the final migration outcome is governed not by the driving force alone, but also by heterostructure geometry, spacer design, defect/vacancy distribution and device configuration.

## 6. Design Rules and Future Perspectives

### 6.1. Interface Sharpness Versus Compositional Grading

The first design question is whether the target device requires a sharp interface or a graded interface. A sharp interface is desirable for stable band alignment, well-defined exciton dissociation and reproducible diode behavior. In contrast, a graded interface may be useful for reducing interfacial strain, smoothing band offsets or creating spatially tunable emission. The same ion-migration process can therefore be either harmful or beneficial depending on the intended function. Future studies should explicitly define the desired interface profile before selecting the fabrication route or post-treatment condition.

### 6.2. In-Plane Versus Out-of-Plane Migration

In-plane and out-of-plane migration should be considered separately because lateral and vertical heterostructures exhibit distinct migration kinetics, interface evolution and reliability concerns. In lateral heterostructures, halide ions mainly migrate along the continuous in-plane inorganic framework or through edge- and defect-assisted pathways, which can lead to relatively fast junction broadening, lateral alloy formation and spatially graded emission. Therefore, lateral heterostructures are useful for studying migration length, interface sharpness and spatially programmable optical responses, but their device reliability is sensitive to interface broadening under thermal or electrical stress. In vertical heterostructures, out-of-plane ion migration is constrained by organic spacer layers and interlayer barriers, so ion transport is generally more confined and slower than in-plane diffusion. However, once the energetic threshold for interlayer exchange is overcome, layer-by-layer alloy formation or interfacial compositional reconstruction may still occur. Thus, vertical heterostructures may provide better interface confinement and device stability for stacked or junction-type devices, but their long-term reliability depends on suppressing cumulative interlayer diffusion and vacancy redistribution. Reporting only a general diffusion phenomenon is therefore insufficient; the migration direction, interface geometry, relative migration speed and dimensional confinement should be stated clearly.

### 6.3. Spacer-Cation Design

Organic spacer cations are key structural variables for regulating ion migration in 2D-OIHP heterostructures. Flexible alkyl spacers generally promote lattice softness and facilitate ion redistribution, whereas rigid aromatic or π-conjugated spacers can suppress halide/vacancy migration by increasing steric hindrance and strengthening organic–inorganic coupling. From a theoretical perspective, vacancy-mediated ion migration is closely related to the energetic cost of vacancy formation and ion hopping. Previous studies have shown that layered perovskites can suppress ion migration by increasing the energy required for ion-vacancy formation, while recent first-principles calculations further indicate that organic spacer cations can modify anionic diffusion pathways and migration energetics in 2D lead-halide perovskite heterostructures [49,56]. Future studies should establish quantitative structure–migration relationships that correlate spacer length, rigidity, polarity, hydrogen bonding capability and hydrophobicity with vacancy formation energies and migration barriers. Importantly, spacer-cation design should also consider charge-transport requirements. Overly insulating spacer layers may suppress ionic migration but simultaneously hinder interlayer electronic coupling and carrier extraction, whereas rationally functionalized spacers may help decouple ionic blocking from efficient charge transport [63,64]. Thus, future spacer engineering should aim to balance ion-migration suppression, carrier mobility and device-specific charge-extraction pathways.

### 6.4. Environmental Effects: Humidity and Oxygen

Environmental factors such as humidity and oxygen should be considered when evaluating ion migration in 2D-OIHP heterostructures. Although organic spacer layers can improve environmental resistance and partially restrict ion transport, ion migration remains possible in 2D lead-halide perovskites, especially under thermal, optical or electrical stimuli [47]. Moisture may penetrate through edges, grain boundaries, surface defects or imperfect heterointerfaces, weakening organic–inorganic interactions and promoting structural distortion, defect formation and halide redistribution [79]. More direct evidence from chiral 2D perovskites has shown that humidity disrupts their structural and chiroptical properties, indicating that water molecules can strongly perturb the organic–inorganic framework even in layered perovskite systems [80]. Oxygen can also accelerate degradation under illumination, as atmospheric exposure has been shown to trigger light-induced degradation in 2D lead-halide perovskites [81]. Although these environmental studies are not all performed specifically on 2D-OIHP heterostructures, their findings indicate that moisture- and oxygen-assisted processes may be particularly detrimental at heterointerfaces, where compositional gradients, strain and defect accumulation already provide favorable sites for ionic redistribution. Therefore, future studies should distinguish intrinsic ion migration from environmentally accelerated migration using controlled-atmosphere, encapsulated and operando measurements.

### 6.5. Quantitative and In Situ Characterization

Many current studies rely heavily on PL imaging and spectral shifts to infer ion migration. Although PL is powerful for visualizing bandgap changes and spatial emission evolution, it does not directly quantify ionic concentration or distinguish ion redistribution from strain relaxation, phase redistribution or defect-related recombination. Direct chemical and operando evidence for ion migration in purely two-dimensional OIHP heterostructures remains limited. Nevertheless, related 2D/3D and lateral 3D/2D perovskite studies provide useful methodological references. For example, cathodoluminescence microscopy combined with XRD and XPS can verify light- or heat-induced spacer-cation and halide migration at 2D/3D interfaces, including PEA^+^ diffusion and Br^−^/I^−^ interdiffusion [82]. Spatially resolved TEM and EDS mapping can confirm anisotropic cation migration and interface evolution in lateral 3D/2D heterostructures [53]. Local electrical and scanning-probe techniques, such as vertical/lateral J–V measurements, C-AFM and KPFM, can correlate ion migration with changes in charge transport, photocurrent and surface photovoltage [83]. More directly, in situ bias TOF–SIMS can track reversible ion redistribution under an applied electric field, providing operando evidence for mobile ions during device operation [84].

Future studies on 2D-OIHP heterostructures should therefore combine PL mapping with complementary chemical, structural, electrical and operando techniques. However, directly comparable Arrhenius-type activation energies for ion migration in different 2D-OIHP heterostructures remain scarce, because many studies report qualitative migration trends, DFT-derived barriers, diffusion behavior or optical/electrical responses rather than standardized experimental activation energies. Quantitative reporting of activation energies, diffusion coefficients, interface widths, ionic concentration profiles, cycling stability and measurement conditions will be essential for establishing a more rigorous mechanistic understanding of ion migration. Advanced operando characterization strategies used in functional nanomaterials, including electrochemical nitrate-reduction catalysts, further highlight the importance of tracking dynamic structural evolution, interfacial reconstruction and composition changes under working conditions [85].

### 6.6. Device-Level Strategies and Challenges

From a device-design perspective, unwanted ion migration may be suppressed by combining several strategies, including the use of rigid and hydrophobic spacer cations, reduction in vacancy concentration through defect passivation, insertion of thin ion-blocking interfacial layers, and optimization of thermal/electrical operating conditions. However, complete ionic blocking should not be achieved at the expense of carrier transport or exciton dissociation. Therefore, future device design should balance ion-migration suppression with efficient electronic transport, for example by selecting spacer or interfacial layers that increase ionic migration barriers while maintaining favorable band alignment and charge-transfer pathways.

### 6.7. Implications for Neuromorphic and Memristive Applications

Beyond conventional optoelectronic devices, controllable ion migration in 2D-OIHP heterostructures may also provide a physical basis for neuromorphic and memristive applications. Reversible or partially reversible redistribution of halide ions and vacancies can modulate interfacial barriers, local band bending and channel conductance, thereby giving rise to history-dependent conductance states, photomemory behavior, synaptic-like plasticity and resistive switching [43,77,86]. In particular, halide perovskites with mixed ionic–electronic transport have been considered promising platforms for adaptive neuromorphic hardware [86], while recent two-dimensional halide perovskite memristor arrays further demonstrate the potential of layered perovskites for programmable memory and neuromorphic functions [77]. Nevertheless, practical applications require precise control over ion-migration volatility, retention time, cycle-to-cycle reproducibility and long-term structural stability.

Overall, future progress requires a transition from simply observing ion migration to deliberately programming ion migration. This transition will depend on coordinated control of interface sharpness, migration direction, spacer-cation design, defect concentration, environmental exposure and operando characterization. By integrating these factors, ion migration may be transformed from an uncontrolled instability factor into a controllable crystal-engineering variable for stable and programmable 2D-OIHP heterostructures.

## 7. Conclusions

This review shows that ion migration in 2D-OIHP heterostructures is governed by the nature of mobile ionic species, external stimuli, migration pathways, spacer-cation design and heterointerface geometry, and can either destabilize interfaces or enable programmable device responses. Therefore, ion migration should be viewed not only as a degradation pathway, but also as an interface-governing and potentially programmable process. The key unresolved challenge is to establish a quantitative, direction-resolved and operando understanding of ionic motion under realistic operating conditions, especially to decouple halide migration, vacancy redistribution, interfacial alloying and local band-structure evolution. Addressing this challenge will require correlated structural, chemical, optical and electrical characterization, together with theoretical modeling of ion-transport pathways and interfacial energy landscapes. Such understanding is essential for determining when ion migration should be suppressed to improve stability and when it can be deliberately programmed for reconfigurable, memory-like or neuromorphic functions in next-generation 2D-OIHP heterostructures.

## Figures and Tables

**Table 1:** Fabrication strategies and initial interface features of 2D-OIHP heterostructures.

Strategy	System	Interface	Process	Initial Interface	Evidence	Ref.
Gas–solid intercalation/ cation exchange	(BA)_2_PbI_4_/(BA)_2_MAPb_2_I_7_	Lateral/vertical	MA^+^ diffusion	Graded interface	PL mapping	[24]
Sequential crystallization	(BA)_2_PbI_4_/(BA)_2_(MA)Pb_2_I_7_	Vertical	Sequential precipitation	Transitional region	Cross-sectional PL	[23]
Halide vapor exchange	RP iodide/Br-exchanged region	Lateral	I^−^/Br^−^ exchange	*n*-dependent interface	PL line scan	[39]
Van der Waals epitaxy	(2T)_2_PbBr_4_/(2T)_2_PbI_4_	Lateral	Edge epitaxy	Relatively sharp interface	Optical/PL images	[44]
Dry transfer/stacking	Stacked 2D-OIHP flakes	Vertical	Mechanical assembly	Abrupt vdW interface	AFM/PL	[40]
Mixed-dimensional assembly	2D-OIHP/CNT	Hybrid	Solution assembly/ transfer	Coupled channel interface	Device response	[43]

**Table 2:** Representative ion-migration behaviors, key observations and functional outcomes in 2D-OIHP heterostructures.

System	Species	Driving Force	Pathway	Interface/Device Outcome	Observations	Ref.
(BA)_2_PbI_4_/(BA)_2_PbBr_4_	I^−^, Br^−^	Heat, gradient	In-plane	Graded alloy interface	PL mapping	[44]
(BA)_2_PbBr_4_/(BA)_2_(MA)_2_Pb_3_I_10_	I^−^, Br^−^	Heat/ concentration gradient	Out-of-plane	Intermediate alloy layer	PL + DFT	[40]
*n*-dependent RP heterostructures	Halides	Halide exchange	Layer- dependent	Low-*n* sharp/high-*n* graded	PL line scan	[39]
BA/PEA/2P systems	Halides, vacancies	Thermal/bias stress	Suppressed migration	Stable sharp interface	PL/device response	[40,61]
(PEA)_2_PbBr_4_/(PEA)_2_PbI_4_	Halides, vacancies	Electric field	Directional	Bias-programmed rectifying junction	I-V/PL	[32]
2D-OIHP/CNT	Mobile ions	Bias, light	Channel- coupled	Photomemory response	Light-tunable I-V	[43]

## Data Availability

No new data were created or analyzed in this study. Data sharing is not applicable to this article.
